# Immigration-Related Factors and Depression Help-Seeking Among Older Chinese Americans

**DOI:** 10.1093/geroni/igab046.570

**Published:** 2021-12-17

**Authors:** Dexia Kong, Man Guo, Melissa Simon, XinQi Dong

**Affiliations:** 1 Rutgers University, New Brunswick, New Jersey, United States; 2 The University of Iowa, Iowa City, Iowa, United States; 3 Northwestern University Feinberg School of Medicine, Chicago, Illinois, United States; 4 Rutgers University, Rutgers Institute for Health, New Jersey, United States

## Abstract

Asian Americans have the lowest mental health service utilization rate among all racial/ethnic groups. One important yet understudied aspect of this group’s mental health service use is its potential associations with immigration-related factors such as migration reasons, years in U.S., acculturation, and ethnic enclave residence. Using data from the Population-based Study of Chinese Elderly in Chicago (collected 2013-2015, N=3,123), this study investigates whether and how immigration-related factors shape mental health service utilization. Four categories of help-seeking behaviors for depressive symptoms were examined, including not seeking help (23.5%), seeking help from informal source(s) only (40%), seeking help from both informal and formal sources (28.7%), and seeking help from formal source(s) only (8.8%). Results of logistic regressions showed that U.S. Chinese older adults who migrated for family reasons were less likely to seek help from informal sources only than those who migrated for other reasons [Odds Ratio (OR)=0.64, 95% Confidence Interval (CI)=0.42-0.99). Less acculturated older immigrants (OR = 0.88, 95% CI = 0.79-0.97) and those who lived in Chinatown (OR = 2.34, 95% CI = 1.21-4.52) were more likely to seek help from formal sources only (relative to not seeking any help). Our findings showed that majority of the older Chinese Americans with depressive symptoms either did not seek help or sought help from informal sources only. Their help-seeking behaviors were shaped by their migration and acculturation experiences. Leveraging informal support networks and ethnicity-specific resources in Chinatown represent a culturally appropriate approach to facilitate mental health help-seeking among U.S. Chinese older adults.

